# Genome-wide annotation and functional identification of aphid GLUT*-*like sugar transporters

**DOI:** 10.1186/1471-2164-15-647

**Published:** 2014-08-04

**Authors:** Daniel RG Price, John A Gatehouse

**Affiliations:** School of Biological and Biomedical Sciences, Durham University, South Road, Durham, DH1 3LE UK

**Keywords:** Facilitative transport, Hexose sugars, Major facilitator superfamily, Membrane transport, Uniport

## Abstract

**Background:**

Phloem feeding insects, such as aphids, feed almost continuously on plant phloem sap, a liquid diet that contains high concentrations of sucrose (a disaccharide comprising of glucose and fructose). To access the available carbon, aphids hydrolyze sucrose in the gut lumen and transport its constituent monosaccharides, glucose and fructose. Although sugar transport plays a critical role in aphid nutrition, the molecular basis of sugar transport in aphids, and more generally across all insects, remains poorly characterized. Here, using the latest release of the pea aphid, *Acyrthosiphon pisum*, genome we provide an updated gene annotation and expression profile of putative sugar transporters. Finally, gut expressed sugar transporters are functionally expressed in yeast and screened for glucose and fructose transport activity.

**Results:**

In this study, using a *de novo* approach, we identified 19 sugar porter (SP) family transporters in the *A. pisum* genome. Gene expression analysis, based on 214, 834 *A. pisum* expressed sequence tags, supports 17 sugar porter family transporters being actively expressed in adult female aphids. Further analysis, using quantitative PCR identifies 4 transporters, *A. pisum* sugar transporter 1, 3, 4 and 9 (*ApST1*, *ApST3*, *ApST4* and *ApST9*) as highly expressed and/or enriched in gut tissue. When expressed in a *Saccharomyces cerevisiae* hexose transporter deletion mutant (strain EBY.VW4000), only ApST3 (previously characterized) and ApST4 (reported here) transport glucose and fructose resulting in functional rescue of the yeast mutant. Here we characterize ApST4, a 491 amino acid protein, with 12 predicted transmembrane regions, as a facilitative glucose/fructose transporter. Finally, phylogenetic reconstruction reveals that ApST4, and related, as yet uncharacterized insect transporters are phylogenetically closely related to human GLUT (SLC2A) class I facilitative glucose/fructose transporters.

**Conclusions:**

The gut enhanced expression of *ApST4*, and the transport specificity of its product is consistent with ApST4 functioning as a gut glucose/fructose transporter. Here, we hypothesize that both ApST3 (reported previously) and ApST4 (reported here) function at the gut interface to import glucose and fructose from the gut lumen.

**Electronic supplementary material:**

The online version of this article (doi:10.1186/1471-2164-15-647) contains supplementary material, which is available to authorized users.

## Background

Phloem feeding insects, which includes aphids, whiteflies, psyllids and planthoppers, feed solely on phloem sap, which contains high concentrations of sucrose (a disaccharide sugar comprising of glucose and fructose). Utilizing sucrose as its main respiratory substrate the pea aphid, *Acyrthosiphon pisum*, catabolizes 15 - 30% of its ingested sucrose in oxidative pathways. A larger proportion, close to 50% of the ingested sucrose, is incorporated into aphid tissues [1, 2]. While sucrose is the main carbon source for *A. pisum*, sucrose itself is not transported across the gut epithelium [3]. Instead sucrose is hydrolyzed in the gut lumen by an α-glucosidase to its constituent monosaccharides, glucose and fructose [4–6], and these hexose sugars are transported. Although sugar transport plays a critical role in aphid nutrition, the molecular basis of sugar transport in phloem feeding insects is poorly characterized. In this study, we address this knowledge gap by testing our prediction that *A. pisum* uses facilitative transporters belonging to the major facilitator superfamily for hexose sugar transport.

The major facilitator superfamily (MFS), one of the largest transporter superfamilies, currently contains 82 families, with each family specific for a group of compounds [7]. Within the MFS, sugars are transported by sugar porter family transporters [transporter classification number (T.C #) 2.A.1.1]. The sugar porter family is essentially ubiquitous and found throughout the tree of life. Currently, our best insight into sugar porter function comes from detailed structure and function analyses of human transporters (reviewed in [8–11]). Human sugar porter family transporters (gene symbol SLC2A, protein symbol GLUT) contain 14 members, which are grouped according to their sequence similarity (GLUTs Class I – III). By far the best-described are GLUT class I transporters (GLUTs 1 – 4 and 14) and GLUT class II transporters (GLUTs 5, 7, 9 and 11), which mediate equilibrative, energy independent membrane transport of hexose sugars down their concentration gradient. GLUT class III transporters (GLUTs 6, 8, 10, 12 and 13) are more recently discovered, as a result of the sequencing of the human genome, and their function is less well-described [8, 11]. Predominant sugars transported by human GLUTs include glucose, galactose, fructose, and myoinositol [8–11].

Previously, using the initial release of the *A. pisum* genome sequence (assembly Acyr_1.0) [12] we identified an expanded family of major facilitator superfamily sugar transporters [13]. The most highly expressed sugar transporter (according to number of gut expressed sequence tags) was named *A. pisum* transporter 3 (ApST3, ACYPI004204). *ApST3* is globally highly expressed in insect tissues and is enriched 2.2-fold in gut tissues, relative to whole insect expression levels [13]. Functionally, ApST3 is a low affinity fructose (*K*_m_ 47 mM fructose) and low affinity glucose (*K*_m_ 66 mM glucose) facilitative transporter [13]. In a related study by Kikuta et al., 18 putative sugar transporters were identified in phloem feeding insect, the rice brown planthopper, *Nilaparvata lugens*. Of these, 6 transporters were highly expressed at the gut interface compared to whole insect levels [14]. The *N. lugens* ortholog of ApST3, designated *N. lugens* sugar transporter 6 (NlST6, BAI83420) has 43% amino acid sequence identity and 60% sequence similarity to ApST3. Transporter *NlST6* is enriched ~9-fold in gut tissues, relative to whole insect expression levels and has an uptake profile similar to ApST3, transporting both glucose and fructose [14]. Based on gene expression pattern, and substrate selectivity, it likely that *A. pisum* ApST3 and the *N. lugens* ortholog NlST6 import glucose and fructose at the gut interface and contribute to carbon nutrition in these phloem feeding insects.

Here, in an effort to more thoroughly describe the extent of *A. pisum* sugar transport at the gut interface we utilize a genome-wide approach to identify sugar transporters and screen for glucose and fructose transport function. In this study, using a *de novo* approach we identify 19 sugar porter family transporters in the latest release *A. pisum* genome (assembly Acyr_2.0, ABLF00000000.2). Gene expression analysis reveals that 4 of these sugar porter family transporters (*ApST1*, *ApST3*, *ApST4* and *ApST9*) are highly expressed at the *A. pisum* gut interface. When functionally expressed in *Saccharomyces cerevisiae* hexose transport deletion mutant (strain EBY.VW4000) only ApST3 (previously described, [13]) and ApST4 (described here) functionally rescue the hexose transport mutant. We report here the detailed functional characterization of *A. pisum* sugar transporter 4 (ApST4, ACYPI0010980). ApST4 is a facilitative glucose and fructose transporter that is structurally, functionally and phylogenetically related to mammalian GLUT class I transporters [8–11]. *ApST4* is globally highly expressed throughout the aphid, enriched 5.9-fold in gut tissues relative to whole insect expression levels, and transports glucose and fructose with high affinity.

## Results

### The A. pisum genome contains a large family of sugar porters

The latest release of the *A. pisum* genome (assembly Acyr_2.0) contains 19 sugar porter family transporters that match the TIGRFAMs sugar porter motif (TIGR00879). All gene annotations are presented in Additional file 1: Table S1. Gene expression analysis, based on 214, 834 *A. pisum* expressed sequence tags (GenBank dbEST release 130101) supports 17 of these sugar porter family transporters being expressed in mixed population adult *A. pisum*. The two remaining transporters (ApST24 and ApST25) are not represented in the *A. pisum* expressed sequence tag (EST) dataset, suggesting that they are not expressed or only expressed at very low levels. One of the expressed transporters, ApST26, is only represented by male ESTs, suggesting that this transporter is male-specific.

The complement of sugar porter family transporters in other insect species included in our analysis (for species information see Methods) ranges between 8 – 28. Comparatively, with 19 predicted sugar porter family transporters, *A. pisum* contains a large family of sugar porters. Only the red flour beetle, *T. castaneum*, with 28 sugar porter family transporters, encodes a larger number of transporters (Additional file 1: Table S2).

### A. pisum sugar porters are highly expressed at the gut interface

**Figure 1:**
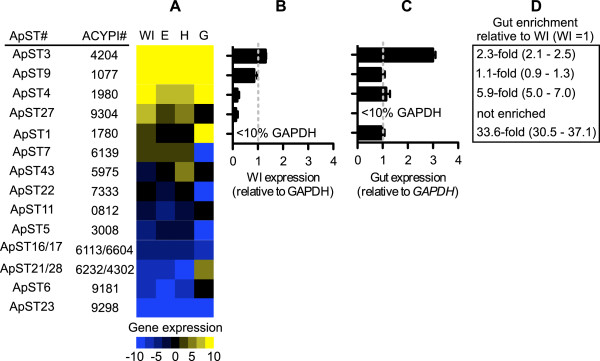
**Gene expression analysis of** ***A. pisum*** **sugar transporters. (A)** Sugar transporter gene expression levels were obtained using real-time quantitative PCR (QPCR) in whole adult females (WI), embryo (E), head (H) and gut (G) tissues. Gene expression was normalized to *GAPDH* and compiled into a heat map, showing high expression (z-score = 10, yellow) to low expression (z-score = −10, blue). Transporter gene expression profiles are ranked from high whole insect (WI) expression (ACYPI# 4204) to low WI expression (ACYPI# 9298). (B and C) Sugar transporter expression level in whole insects **(B)** and gut tissue **(C)** relative to *GAPDH* (*GAPDH* = 1, grey dashed line). Only transporters >10% *GAPDH* expression are shown (*n* = 3, with error bars representing ± SEM). **(D)** Transporters enriched > 2-fold in gut tissue relative to whole insect (WI) are indicated with confidence limits [15] in parentheses (*n* = 3). Transporter identification numbers (ApST#) are as previously described [13]. All transporter sequences are available from NCBI by appending ACYPI00 to each of the ACYPI#.

Quantitative gene expression analysis of *A. pisum* sugar porter family transporters reveals that four transporters: *ApST3*, ACYPI004204; *ApST4*, ACYPI001980; *ApST9*, ACYPI001077; and *ApST27*, ACYPI009304, are highly expressed in adult females. Sugar porter family transporter expression is ranked *ApST3* > *ApST9* > *ApST4* > *ApST27*, in adult females, with the expression of all transporters above our >10% *GAPDH* expression cut-off level (Figure 1A and B). Notably, three of these transporters (*ApST3*, *ApST4*, *ApST9*) are also the most highly expressed sugar porter transporters in *A. pisum* gut tissue. A fourth transporter *ApST1*, ACYPI001780, is expressed at low levels in whole insects (<10% *GAPDH*), and is highly expressed (>10% *GAPDH*) in *A. pisum* gut tissue (Figure 1C). Based on their differing expression profiles we can divide these four gut-expressed sugar porter family transporters into two groups; the first group includes *ApST1*, which has a strongly gut-bias expression pattern (up 33-fold in gut tissue relative to whole insect expression) (Figure 1D). The second group contains transporters *ApST3*, *ApST4* and *ApST9* that are universally highly expressed in whole adult female *A. pisum* and all tissues analyzed including embryos, heads and guts (Figure 1A). As well as being globally highly expressed, transporters *ApST3* and *ApST4* are enriched in gut tissue 2.3-fold and 5.9-fold, respectively, compared to whole insect expression levels (Figure 1C). Although highly expressed in all tissues analyzed, transporter *ApST9* is not enriched in gut tissue, relative to whole insect expression levels (Figure 1C).

### A. pisum gut expressed sugar porters transport hexose sugars

All four *A. pisum* gut expressed sugar transporters (*ApST3* from our previous study [13] and *ApST1*, *ApST4* and *ApST9* reported here) were screened for hexose transport activity by functional complementation of the *Saccharomyces cerevisiae* hexose-uptake deficient strain EBY.VW4000 [16].

**Figure 2:**
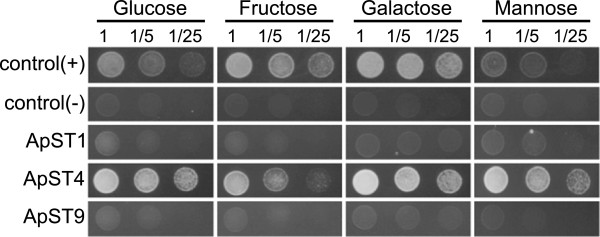
**Functional identification of** ***A. pisum*** **glucose and fructose transporters.** *Saccharomyces cerevisiae* hexose transport mutant EBY.VW4000 was transformed with *A. pisum* gut expressed sugar transporters ApST1, ApST4 and ApST9. Positive control cells (+) were transformed with ApST3 [13] and negative control (−) cells were transformed with empty expression vector. Yeast cell suspensions containing 10 μl of 1, 1/5 and 1/25 OD_600_ units were plated on minimal media containing 60 mM sugars (as indicated) as the sole carbon source. Recovery of mutant yeast growth was observed after 3-days at 30°C.

ApST3, a low affinity fructose/glucose transporter [13] was used as a positive control, and run alongside the functional screen of ApST1, ApST4 and ApST9. Yeast cells transformed with either *ApST3* (positive control from [13]), *ApST1*, *ApST4* or *ApST9* expression constructs produced recombinant aphid transporter protein, which was detected in yeast total membrane fractions by anti-myc antibodies in western blots (Additional file 1: Figure S1). All aphid sugar transporters reported here (including the positive control ApST3) were detected below their calculated molecular weight when expressed in yeast (Additional file 1: Figure S1). The anomalous migration of transmembrane transporters on SDS-PAGE gels is attributed to the hydrophobic properties of the protein [17]. Immunoreactivity was not present in negative control cells that were transformed with an empty pDR195 expression construct (Additional file 1: Figure S1). Although all *A. pisum* transporters were expressed, only ApST3 (positive control from [13]) and ApST4 were able to restore EBY.VW4000 growth on minimal media containing glucose, fructose, galactose and mannose as the sole carbon source (Figure 2). Importantly, negative control yeast cells (transformed with empty pDR195 expression vector) were unable to grow on minimal media plates containing these sugars (Figure 2). These results show that transporters ApST3 (as reported previously [13]) and ApST4 are functional hexose transporters that mediate the efficient transport of hexose sugars across the yeast plasma membrane.

### ApST4 is a facilitative hexose transporter

**Figure 3:**
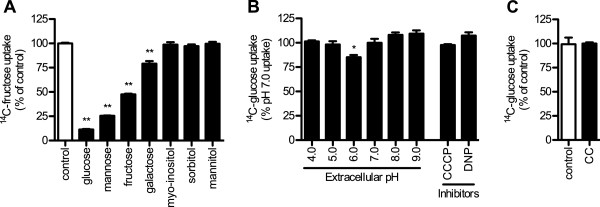
**Functional characterization of ApST4 in whole yeast cells.** Transport specificity of ApST4 was investigated by functional expression in EBY.VW4000 yeast cells (EBY-ST4 cells). **(A)** Inhibition of ^14^C-fructose uptake (50 mM initial extracellular concentration) in competition assays containing 100 mM unlabeled sugars (as indicated), uptake is displayed as percentage of no competing sugar (control). **(B)** pH dependence of ^14^C-glucose uptake in EBY-ST4 cells using indicated extracellular pH; and in the presence of transport inhibitors: 1 μM CCCP (Carbonyl cyanide 3-chlorophenylhydrazone) and 1 μM DNP (2,4-Dinitrophenol). All uptake is displayed as percentage pH 7.0 transport. **(C)** Na^+^ dependence of ^14^C-glucose uptake in EBY-ST4 cells using standard uptake buffer with addition of 50 mM NaCl (control), or in similar buffer were NaCl is replaced with an equimolar amount of choline chloride (CC). All uptake experiments were performed at 30°C and cells were collected by rapid-filtration after 10 min. Sugar uptake was determined by liquid scintillation counting. Transport was corrected for background uptake into control (−) cells transformed with an empty expression vector. Each value is the mean ± SEM, *n* = 3. Bars marked with asterisks are significantly different from controls. *P < 0.01 and **P < 0.001; one way-ANOVA followed by Dunnett’s post-test.

ApST4 is a broad-spectrum hexose transporter. Substrate competition (inhibition) experiments with a 2-molar excess (100 mM initial extracellular concentration) of unlabeled competing sugar, demonstrate that glucose, mannose, fructose and galactose significantly reduce ^14^C fructose uptake into yeast cells expressing ApST4 (*P* < 0.001, one-way ANOVA followed by Dunnett’s posttest, Figure 3A). ApST4 efficiently binds glucose > mannose > fructose, all reducing ^14^C-fructose uptake by > 50% compared to control uptake with no competing sugar. Galactose also competes with fructose uptake, but less efficiently, only reducing ^14^C-fructose uptake by ~21% compared to control uptake with no competing sugar (Figure 3A). Competition assays with alcohol sugars: myo-inositol, sorbitol and mannitol revealed these do not compete with ^14^C-fructose uptake, and are therefore not recognized substrates (Figure 3A). In summary, functional complementation assays (Figure 2) and competition (inhibition) assays (Figure 3A) are consistent with ApST4 functioning as a broad-spectrum hexose transporter.

Major facilitator superfamily (MFS) transporters function as either facilitative transporters or secondary active transporters. Facilitative transporters (uniporters) mediate passive (equilibrative) solute transport across membranes, moving solutes along their concentration gradients with no energy expenditure. In contrast, secondary active transporters (symporters) couple solute transport with monovalent cation (either H^+^ or Na^+^) transport. Symporters require an electrochemical membrane potential to drive transport, allowing solutes to accumulate against their concentration gradient [10, 18, 19]. ApST4 transports glucose over a wide extracellular pH range, between pH 4.0 and 9.0 (Figure 3B) and pre-treatment of yeast cells expressing ApST4 with proton ionophores carbonyl cyanide 3-chlorophenylhydrazone (CCCP) and 2,4-Dinitrophenol (DNP) does not significantly reduce glucose transport compared to untreated control cells (Figure 3B). Furthermore, there was no significant effect on ApST4 transport activity when NaCl in the transport buffer was substituted with choline chloride (Figure 3C). Our data demonstrates that ApST4 is a facilitated hexose transporter (uniporter) that functions independently of the electrochemical membrane potential.

**Figure 4:**
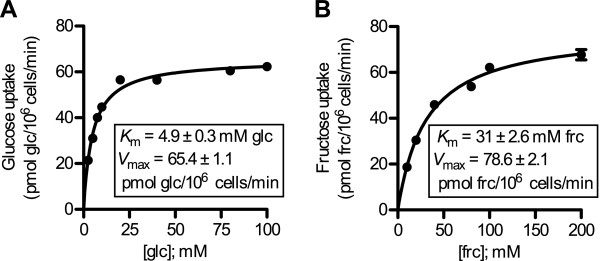
**Glucose and fructose uptake kinetics of ApST4.** Yeast cells expressing ApST4 were incubated in various concentrations of ^14^C-glucose and ^14^C-fructose (as indicated). **(A)** Glucose and **(B)** fructose uptake kinetics were fitted to Michaelis-Menten equations and *K*_m_ and *V*_max_ was determined by nonlinear regression using Prism 5.0c software. All uptake experiments were performed at 30°C and cells were collected by rapid-filtration after 10 min. Sugar uptake was determined by liquid scintillation counting. Transport was corrected for background uptake into control (−) cells transformed with an empty expression vector. Each value is the mean ± SEM, *n* = 3.

Glucose and fructose uptake by ApST4 is concentration dependent, with transport rates saturating at high substrate concentrations, yielding an estimated *K*_m_ of 4.9 ± 0.3 mM for glucose and 31.0 ± 2.6 mM for fructose (Figure 4A and B).

### ApST4 shares sequence features with mammalian GLUT facilitative transporters

*ApST4* (full-length gene: 2066 bp) was reconstructed from sequence reads FF306288 and FF306499, present in the *A. pisum* full-length cDNA resource [20]. *ApST4* comprises of a 403 bp 5′ UTR, a 1476 bp coding sequence and a 185 bp 3′ UTR that contains a putative polyadenylation signal (UAUAAA) 22 bp upstream from the poly-A tail. *ApST4* contains 9 exons that map to a 40.8 kbp region of scaffold 735 (total size 263.6 kbp) from *A. pisum* genomic scaffolds of assembly Acyr 2.0. Expression data retrieved from NCBIs dbEST, shows that *ApST4* is expressed in all *A. pisum* sexual morphs including males (10 ESTs), sexual oviparous females (6 ESTs) and asexual females (7 ESTs). Partial sequence orthologs of *ApST4* are present in the green peach aphid, *Myzus persicae* (3 ESTs); cotton aphid, *Aphis gossypii* (2 ESTs) and brown citrus aphid, *Toxoptera citricida* (1 EST).

**Figure 5:**
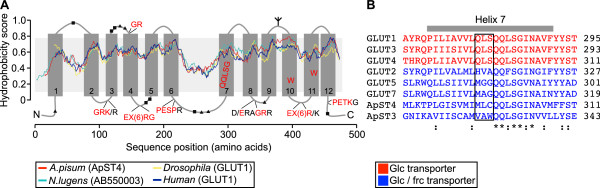
**Sequence analysis of ApST4 and related GLUT transporters. (A)** Alignment of hydrophobicity profiles for ApST4 (ACYPI001980) and GLUT class I transporters from: *Nilaparvata lugens* (AB550003); *Drosophila melanogaster* (CG1086); and *Homo sapiens* (GLUT1). Sequence position (in amino acids) and Kyte and Doolittle hydrophobicity score (normalized between 0–1) is shown on the x-axes and y-axes, respectively. Membrane topology of ApST4 is shown with transmembrane (TM) helices 1 – 12 predicted by TMHMM (http://www.cbs.dtu.dk/services/TMHMM/). Conserved sugar porter family amino acid motifs are highlighted and ApST4 residues that match the motif are coloured red. Predicted ApST4 post-translational modifications include: N-glycosylation (ψ); and phosphorylation at serine (∎) and threonine (▲) residues. **(B)** Sequence alignment of TM7 of selected GLUT transporters. TM7 from human glucose transporters (GLUTs 1, 3 and 4); human glucose/fructose transporters (GLUTs 2, 5 and 7); and *A. pisum* glucose/fructose transporters ApST3 (ACYPI004204) and ApST4 (ACYPI001980) are shown. Sequences were aligned using the ClustalX program, and ordered according to their similarity. Boxed regions indicate location of the QLS substrate binding motifs. Asterisks indicate identical residues in all sequences; colon indicates conservative amino acid substitution; dot marks semi-conservative amino acid substitution.

The ApST4 protein encoded by the longest open reading frame (ORF) comprises 491 amino acids, with a predicted molecular weight of 53.4 kDa. ApST4 belongs to major facilitator superfamily (MFS), sugar porter (SP) family (T.C # 2.A.1.1) and has 12 predicted transmembrane α-helices (as predicted by TMHMM, http://www.cbs.dtu.dk/services/TMHMM/), typical of related mammalian and insect GLUT transporters (Figure 5A). ApST4 shares 36% – 45% sequence identity, and 50% – 58% similarity, with human GLUT class I transporters (GLUTs 1 – 4), with strong sequence conservation in the transmembrane helix regions (Additional file 1: Figure S2). Both ApST3 [13] and ApST4 (reported here) contain signature sequences that are conserved across GLUT family members (Figure 5A and B). Conserved residues in transmembrane helix 7 (TM7) that are critical for substrate binding [9, 21] are consistent with ApST3 and ApST4 functioning as glucose and fructose transporters (Figure 5B).

### ApST4 is phylogenetically related to mammalian GLUT transporters

**Figure 6:**
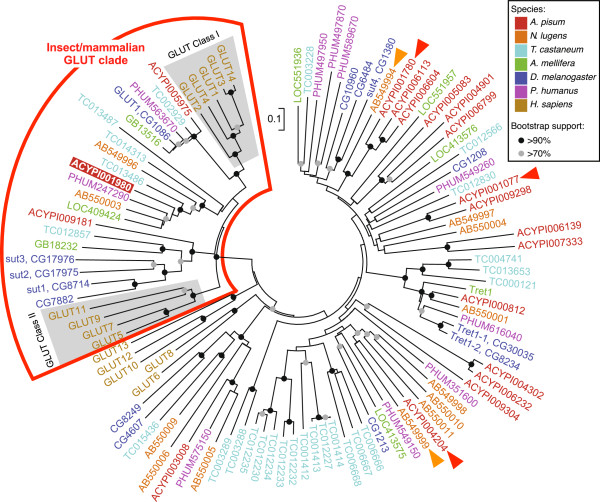
**Phylogenetic analysis of aphid GLUT-like sugar transporters.** Neighbor-joining phylogenetic tree of GLUT-like transporters from *A. pisum* (red), *N. lugens* (orange), *T. castaneum* (light blue), *A. mellifera* (green), *D. melanogaster* (blue), *P. humanus* (purple) and *H. sapiens* (brown). Putative sugar transporters identified by the presence of a TIGRFAMs sugar porter motif (TIGR00879) were included in the analysis. The reliability of the tree was determined using 1000 bootstrap replications. Bootstrap values >90% (black circle) and >70% (grey circle) are indicated at each node. The phylogenetically well-supported insect/mammalian GLUT clade (red outline) contains aphid glucose/fructose transporter ApST4, ACYPI001980 (red box) and human GLUT class I and II transporters (shaded grey). Red arrows indicate aphid gut expressed transporters: ApST1, ACYPI001780; ApST3, ACYPI004204 [13]; and ApST9, ACYPI001077. Orange arrows indicate functionally characterized *N. lugens* sugar transporter transporters (NlST): NlST1, AB549994 [14, 23] and NlST6, AB54999 [14]. All sequences are available from NCBI using the accession numbers in the phylogenetic tree. Scale bar represents 0.1 substitutions per amino acid site.

The phylogenetic position of ApST4 was investigated by comparison with related transporters from humans and other insects. Insect taxa, including *N. lugens*, *T. castaneum*, *A. melifera*, *D. melanogaster*, *P. humanus* were chosen to represent the insect orders: Hemiptera, Coleoptera, Hymenoptera, Diptera, and Phthiraptera, respectively. Phylogenetic reconstruction places APST4 in a well-supported clade containing unknown function insect transporters; mammalian GLUT class I transporters (GLUTs 1–4 and 14); and GLUT class II transporters (GLUTs 5, 7, 9, 11) (Figure 6) [22]. ApST4 (ACYPI001980) is most closely related to single orthologous insect transporters, all of unknown function, from *P. humanus* (PHUM247290), *A. mellifera* (LOC409424), and multiple (one to many) orthologs in *T. castaneum* (TC013486, TC014313 and TC013487) and *N. lugens* (AB550003 and AB549996). ApST4 and closely related (as yet uncharacterized) insect transporters are more distantly related to well-characterized mammalian GLUT class I facilitative hexose transporters (Figure 6) [22].

## Discussion

Here, in an update to our original sugar transporter gene annotation [13], we have used a *de novo* search to identify 19 sugar porter family transporters in the latest version of the *A. pisum* genome (assembly Acyr_2.0). Compared to our previous study, which identified 29 putative sugar transporters [13], we present a subset of the original gene list (19/29 transporters) that match the TIGRFAMS sugar porter family motif (TIGR00879). The refinement of the original gene set is due in part, to a more stringent cut-off value used for transporter identification, combined with five gene models being withdrawn from the latest *A. pisum* genome assembly. The withdrawn gene models were previously identified as: ApST10, ACYPI007611; ApST33, ACYPI007211; ApST41, ACYPI006926; ApST51, ACYPI003842; and ApST55, NW_001921974 [13]. A reduced number of putative sugar transporter genes were also identified in *de novo* searches of other insect genomes (Additional file 1: Table S2). This reduction is again due to use of a more stringent cut-off used for whole genome *de novo* searches combined with use of updated genome assemblies.

Of the 19 annotated *A. pisum* sugar porter family transporters (matching the TIGRFAMs sugar porter family motif TIGR00879), four transporters: ApST1, ApST3, ApST4 and ApST9 are highly expressed and/or enriched in gut tissue. In our functional assays of these four transporters, only ApST3 (as reported previously [13]) and ApST4 (reported here) transport glucose and fructose. Our data is consistent with *A. pisum* using facilitative transporters ApST3 and ApST4 at the gut interface for import of glucose and fructose [3, 5]. Based on transport function, we hypothesize that glucose and fructose moving passively from high concentrations in the gut lumen, to low concentrations of glucose and fructose in the haemolymph [24]. The functional role of ApST1 and ApST9 at the *A. pisum* gut interface remains unknown. Although ApST1 and ApST9 were expressed in the yeast hexose-transport deficient mutant EBY.VW4000 (Additional file 1: Figure S1), they both failed to functionally complement the yeast mutant (Figure 2). It is possible that ApST1 and ApST9 were not efficiently targeted to the yeast plasma membrane and were not available for sugar uptake. Alternatively, the hexose sugars used in our functional assays were not recognized substrates. Current work is underway to functionally express ApST1 and ApST9 and further investigate their transport properties and substrate selectivity.

Aphid transporters ApST3 and ApST4 are structurally, functionally and phylogenetically related to mammalian GLUTs. Both ApST3 and ApST4, like their human GLUT orthologs have a conserved sequence motif in transmembrane helix 7 (TM7) that is critical for substrate selectivity [9, 21, 25] (Figure 5 and Additional file 1: Figure S3). Specifically, human GLUTs 1, 3 and 4, which transport glucose but not fructose have a conserved QLS amino acid motif in TM7; whereas GLUTs 2, 5, 7, 9 and 11 which transport glucose and fructose have amino acid residues: HVA, MGG, MAG, MAC, or GSA respectively, in the equivalent position [9, 21, 25] (Figure 5B and Additional file 1: Figure S3). The functional role of the QLS motif for determining glucose specificity has been experimentally validated in two independent experiments [21, 25]. First, a GLUT3/2 chimera, where the beginning of TM7 to the C-terminus of GLUT3, was replaced by the corresponding region of GLUT2, converted the protein to a glucose/fructose transporter, with GLUT2 kinetics [25]. Second, a substitution experiment, where GLUT2 had amino acids HVA replaced with QLS, and GLUT3 had amino acid QLS replaced with HVA resulted in partial reversal of their substrate specificities and uptake kinetics [21]. Insect GLUT-like transporters ApST3 [13], ApST4 (reported here) and NlST6 [14] are all facilitative glucose/fructose transporters. Sequence analysis, demonstrates that these transporters, similar to human GLUT glucose/fructose transporters, do not have a QLS-motif in TM7, consistent with glucose and fructose transport (Figure 5B and Additional file 1: Figure S3). In an extension of this observation, based on the presence of the QLS amino acid motif within TM7 for all insect GLUT class I orthologs included in our analysis (*A. pisum*, ACYPI005975; *T. castaneum*, TC002929; *P. humanus*, PHUM563670; *D. melanogaster*, CG1086 and *A. mellifera*, GB13516) (Additional file 1: Figure S3), we make the prediction that they are all specific for glucose (and not fructose). Current work is underway to further investigate structure/function relationships across insect GLUT class I orthologs.

Our functional screen specifically targeted sugar transporters belonging to the major facilitator superfamily, the largest family of characterized sugar transporters. However, it is possible that aphid transporters belonging to the solute:sodium symporter (SSS) family [transporter classification number (T.C #) 2.A.21] also transport sugars. As with the sugar porter family, previously described, sugar transporters within the solute:sodium symporter family are also most extensively characterized in humans (Reviewed in [26]). Human sodium-glucose transporters, also known as Na^+^/glucose cotransporters (gene symbol SLC5A, protein symbol SGLT), mediate active transport of glucose *i.e.* they can move glucose against its concentration gradient. The *A. pisum* genome contains 7 solute:sodium symporter family transporters (Pfam sodium:solute symporter motif, PF00474), 6 of which are predicted to function as sodium/monocarboxylate cotransporters, and the other as a sodium/choline cotransporter (Additional file 1: Table S3). Importantly, our whole genome *de novo* searches did not identify *A. pisum* solute:sodium symporter family glucose transporters. Therefore, our data predicts that *A. pisum* uses only facilitative sugar transporters for sugar homeostasis, and not as in mammals, a combination of facilitate transporters and active transporters [10, 11, 26]. More generally, we hypothesize that aphid sugar homeostasis is energy-independent and maintained by facilitated diffusion (downhill movement) of sugars. This prediction necessitates a large family of related sugar transporters, with differing tissue expression patterns, and importantly subtle differences in substrate affinity between transporters.

## Conclusions

In summary, this study demonstrates that *A. pisum* encodes a large family of sugar porter family transporters. Based on gene expression patters, only 4 transporters (ApST1, ApST3, ApST4 and ApST9) are predicted to play a functional role at the gut interface. Here we characterize *A. pisum* sugar transporter 4, ApST4, as a facilitative glucose/fructose transporter. Our gene expression and functional transport analyses are consistent with ApST4 playing a role in aphid sugar transport, functioning at the gut interface to transport glucose and fructose. Here, we demonstrate that ApST4 is, structurally, functionally and phylogenetically related to mammalian GLUT class I facilitative transporters. By placing transporters in a phylogenetic framework this work will facilitate prediction and testing of as yet uncharacterized sugar transporters across related taxa.

## Methods

### Aphid culture

Parthenogenetic females of the pea aphid, *Acyrthosiphon pisum* (Harris), lineage UY2 [27] were maintained on pre-flowering *Vicia faba* cv. The Sutton at 20°C in a long day regime of 16 h of light and 8 h of dark.

### De novo identification of sugar transporters

The current version of the *A. pisum* genome Acyr_2.0 (GenBank assembly ID: GCA_000142985.2) contains 16932 gene models and 17718 protein models. Putative *A. pisum* sugar transporters matching the TIGRFAMs sugar porter (SP) family motif (TIGR00879 [28]) were identified in the latest *A. pisum* reference protein dataset using the hmmsearch program, which is part of the HMMER package (version 3.0) [29]. All identified sugar porter family transporters had a hmmsearch sequence score >237.80 (trusted cutoff).

For comparison, sugar porter family transporters were identified in a partial reference protein datasets from *Nilaparvata lugens* [30, 31]; the reference protein datasets derived from whole genome sequence of *H. sapiens;* and whole genome sequences of 6 insect species. The insect species included in our comparative analysis were: *Anopheles gambiae*, *Apis mellifera*, *Drosophila melanogaster*, *Nasonia vitripennis*, *Pediculus humanus*, and *Tribolium castaneum.* Protein reference sequences used in the analysis are available from Ensembl Genomes (http://ensemblgenomes.org/) and the version of the genome assembly used for the HMMER searches is listed in Additional file 1: Table S2. All identified sugar porter family transporters had a hmmsearch sequence score >237.80 (trusted cutoff). Following *de novo* identification of sugar transporters, the phylome database (phylomeDB, http://phylomedb.org/), which is a complete phylogenetic analysis whole genomes [32] was searched for any additional homologs.

### Real-time quantitative PCR

Real-time quantitative PCR (QPCR) was used to compare the gene expression profile of 16 *A. pisum* sugar porter family transporters in different tissues using 2^-ΔΔ*C*T^ methodology [15]. All identified sugar porter family transporters were included, except ApST24, ApST25 and ApST26, which were excluded from the analysis due to the absence of supporting ESTs in whole adult female *A. pisum*. Primers were designed using Primer express software for real-time PCR v3.0 (Applied Biosystems) and specificity was checked using Primer-Blast (available at NCBI) against the *A. pisum* Refseq gene models and Acyr_2.0. Primer sequences are shown in Additional file 1: Table S4. For comparative gene expression analysis QPCR primers had amplification efficiencies > 90% and < 105%. All experiments were performed in triplicate including no template controls and no reverse-transcription controls. Each reaction comprised of 1X Power SYBR green master mix (Applied Biosystems), 200 nM of forward and reverse primers and cDNA derived from 8 ng total RNA from whole insect, head, gut and embryos. Sugar transporter gene expression was normalized to *glyceraldehyde-3-dehydrogenase* (*GAPDH*, ACYPI009769) in each tissue. In *A. pisum* we have established *GAPDH* as a reliable housekeeping gene to normalize gene expression as it is uniformly expressed throughout aphid development and across aphid tissues [13, 33, 34]. Normalized aphid transporter gene expression was and compiled into a heat map (z-score × 10, [z = (each value – mean)/standard deviation]). Yellow: z > 0 (high expression), blue z < 0 (low expression) and black z = 0.

### ApST expression constructs and yeast transformation

Full-length coding sequences for gut expressed sugar transporters *ApST1*, *ApST4*, and *ApST9* were amplified from whole adult female *A. pisum* cDNA using Phusion proof-reading polymerase (Finnzymes). All PCR primers contained a 5′ optimized Kozak initiation sequence for efficient translation in yeast [35] and either a 5′ XhoI or EcoRV site and a 3′ NotI site (primer sequences are shown in Additional file 1: Table S5). Amplified coding sequences of *ApST1* and *ApST4* were digested with XhoI and NotI and *ApST9* was digested with EcoRV and NotI and cloned into the respective sites of the yeast shuttle vector pDR195, previously modified to encode a 3′ c-Myc epitope [13]. The *ApST3* yeast expression construct, with a 3′ c-Myc epitope, was constructed previously, as described by [13]. *ApST* expression constructs were fully sequenced and used to transform *Saccharomyces cerevisiae* hexose transporter deletion mutant EBY.VW4000 [16] by the lithium acetate/PEG method [36]. Due to deletion of at least 20 hexose transporters, EBY.VW4000 has very low hexose transport activity, and is unable to grow on minimal media plates containing hexose sugars as the sole carbon source [16]. Transformants were selected on synthetic complete (SC) media, pH 5.6 (0.17% yeast nitrogen base, 2% maltose, 1% agar supplemented with uracil drop-out mix) at 30°C for 3–4 days. Recombinant transporter expression was detected by screening total membrane proteins from transformed cells by western blot using anti-c-Myc antibodies, as described in [13]. Positive transformants were replica plated on SC media lacking maltose but containing 60 mM glucose, fructose, galactose or mannose and recovery of EBY.W4000 growth was assessed after 3 days at 30°C. Controls were run in parallel and consisted of cells transformed with either the ApST3 fructose/glucose transporter [13] (positive control), or empty pDR195 vector (negative control).

### ApST uptake assays in whole yeast cells

Uptake assays in whole yeast cells were based on methods described by [37]. Briefly, yeast cells were grown overnight at 30°C in liquid SC media containing 2% maltose, cells were harvested and washed twice in ice-cold transport buffer (50 mM NaPO_4_ buffer pH 7.0) and resuspended to 30 OD_600_/ml in the same buffer (unless indicated differently). For standard uptake assays, cells were incubated in a rotary shaker at 30°C and uptake assays were initiated by adding defined hexose sugars (50 mM initial extracellular concentration) labeled with D-[U-^14^C]-glucose (Perkin-Elmer) or D-[U-^14^C]-fructose (Perkin-Elmer), depending on the experiment. At given intervals 2 OD of cells were collected by rapid filtration onto nitrocellulose membranes (0.2 μm pore size) under vacuum and washed with an excess of ice-cold distilled water. Transport of ^14^C-glucose or ^14^C-fructose into yeast cells was determined by liquid scintillation counting. Glucose and fructose transport into yeast cells expressing ApST4 increased linearly for at least 30 min. Thus, all uptake assays were stopped after 10 min in the linear phase of uptake. Sugar selectivity experiments were performed by adding a 2-fold molar excess of unlabeled sugar (100 mM initial extracellular concentration) to the standard ^14^C-labeled fructose uptake assay. pH dependence was analyzed by performing uptake assays in either 50 mM citrate buffer (pH 4 – 6), or 50 mM phosphate buffer (pH 7 – 9), as indicated. The influence of pH gradient was determined by preincubating cells for 5 min with either 1 μM 2,4-dinitrophenol (DNP) or 1 μM carbonyl cyanide 3-chlorophenylhydrazone (CCCP). Sodium ion (Na^+^) dependence was analyzed by performing standard uptake assays with the addition of 50 mM NaCl to the transport buffer, control experiments were run in parallel, where NaCl was replaced with an equimolar amount of choline chloride. Kinetic parameters *K*_m_ and *V*_max_ were estimated by fitting uptake rates at various substrate concentrations (as indicated) to the Michaelis–Menten equation using non-linear regression (Prism vs. 5.0c software, GraphPad). All uptake assays were performed in triplicate, using an independent yeast clone for each replicate and uptake data were corrected for background uptake into cells transformed with an empty pDR195 vector.

### Sugar transporter sequence analysis and phylogenetic analysis

Sequence features in sugar transporter proteins were predicted using online search engines. Specifically, transmembrane α-helix regions and membrane topology was predicted with the transmembrane using hidden Markov models (TMHMM) server (available at http://www.cbs.dtu.dk/services/TMHMM/) [38], or alternatively using Kyte and Doolittle hydrophobicity analysis (available at http://web.expasy.org/protscale/) [39]. Serine/threonine phosphorylation sites were predicted using the NetPhos 2.0 server (available at http://www.cbs.dtu.dk/services/NetPhos/) [40]. Transporter N-linked glycosylation sites were predicted using NetNGlyc 1.0 server (available at http://www.cbs.dtu.dk/services/NetNGlyc/).

For phylogenetic analysis, protein sequences were aligned using MUSCLE [41] and ambiguously aligned positions were excluded by trimAL v1.3 [42]. Neighbour joining (NJ) phylogenetic trees, with 1000 bootstrap replicates, were constructed using MEGA5.2.2 [43].

### Availability of supporting data

All data for the phylogenetic analysis (presented in Figure 6), which includes: sugar porter (SP) family protein sequences; MUSCLE aligned protein sequences; and the neighbor-joining phylogenetic tree are publically available in the LabArchives repository [22].

## Electronic supplementary material

Additional file 1: Figure S1: Western blot analysis of recombinant A. pisum sugar transporters expressed in yeast. **Figure S2.** Sequence alignment and transmembrane predictions for human GLUT class I transporters (GLUTs 1 - 4) and related insect transporters from: *Acyrthosiphon pisum, Drosophila melanogaster* and *Nilaparvata lugens*. **Figure S3.** Sequence alignment of transmembrane 7 (TM7) for human GLUT class I transporters (GLUTs 1-4 and 14), class II transporters (GLUTs 5, 7, 9 and 11) and insect orthologs. **Table S1.** Summary of annotated *A. pisum* sugar porter family transporters. **Table S2.** *de novo* identification of sugar porter (SP) family transporters [transporter classification number (T.C #) 2.A.1.1] in insect genomes. **Table S3.** Summary of annotated *A. pisum* solute:sodium symporter (SSS) family transporters. **Table S4.** Quantitative PCR primers for *A. pisum* sugar transporters and housekeeping gene glyceraldehyde-3-phosphate dehydrogenase (*GAPDH*). **Table S5.** *A. pisum* sugar transporter coding sequence primers for *Saccharomyces cerevisiae* expression constructs. (DOCX 2 MB)
