# Supplementary material for: Genome-wide annotation and functional identification of aphid GLUT-like sugar transporters
Source: BMC Genomics. 2014 Aug 4;15(1):647. doi: 10.1186/1471-2164-15-647 (PMC4132908; doi:10.1186/1471-2164-15-647)
Supplement: Supplementary file 1 — Additional file 1: Figure S1: Western blot analysis of recombinant A. pisum sugar transporters expressed in yeast. Figure S2. Sequence alignment and transmembrane predictions for human GLUT class I transporters (GLUTs 1 - 4) and related insect transporters from: Acyrthosiphon pisum, Drosophila melanogaster and Nilaparvata lugens. Figure S3. Sequence alignment of transmembrane 7 (TM7) for human GLUT class I transporters (GLUTs 1-4 and 14), class II transporters (GLUTs 5, 7, 9 and 11) and insect orthologs. Table S1. Summary of annotated A. pisum sugar porter family transporters. Table S2. de novo identification of sugar porter (SP) family transporters [transporter classification number (T.C #) 2.A.1.1] in insect genomes. Table S3. Summary of annotated A. pisum solute:sodium symporter (SSS) family transporters. Table S4. Quantitative PCR primers for A. pisum sugar transporters and housekeeping gene glyceraldehyde-3-phosphate dehydrogenase (GAPDH). Table S5. A. pisum sugar transporter coding sequence primers for Saccharomyces cerevisiae expression constructs. (DOCX 2 MB) [file 12864_2014_6344_MOESM1_ESM.docx]

**Additional File 1**

**Figure S1. Western blot analysis of recombinant *A. pisum* sugar transporters expressed in yeast.** Western blot analysis of total membranes (5 μg membrane protein/lane) from *Saccharomyces cerevisiae* hexose transport mutant EBY.VW4000 expressing ApST1, ApST4, ApST9. Positive control cells (+) were expressing ApST3 (previously reported in [1]); and negative control cells (-) were transformed with empty pDR195 expression vector. Yeast membrane proteins were separated on a 12.5% polyacrylamide gel, transferred to nitrocellulose, probed with anti-c-Myc antibodies and exposed to film. The signal intensities from each lane are directly comparable. Calculated molecular weight (kDa) for each transporter protein is shown in boxes.

**Figure S2. Sequence alignment and transmembrane predictions for GLUT class I transporters.** Human GLUT class I transporters (GLUTs 1 - 4) and related transporters from: *Acyrthosiphon pisum*, *Drosophila melanogaster* and *Nilaparvata lugens* are shown. Sequences were aligned using the ClustalX program, and ordered according to their similarity. Membrane spanning regions (Helix 1 – 12) for each transporter were predicted using the TMHMM program (http://www.cbs.dtu.dk/services/TMHMM/) and shaded grey. Boxes highlight conserved Gln^177^ and the ^295^MLC motif of ApST4 (ACYPI001980). Asterisks, identical residues in all sequences; colon, conservative amino acid substitution; dot, semi-conservative amino acid substitution.

**Figure S3. Sequence alignment of TM7 of human GLUT class I and II transporters and insect orthologs.** TM7 from human GLUT class I transporters (GLUTs 1-4 and 14); GLUT class II transporters (GLUTs 5, 7, 9 and 11) and all insect orthologs are shown. Sequences from TM7 of all transporters were aligned using the ClustalX program. Boxed regions indicate location of the QLS substrate binding motifs. Functionally characterized transporters are shaded grey and substrate specificities are shown. Transported sugars are abbreviated: frc, fructose; gal, galactose; glc, glucose; man, mannose; myo-ins, myo-inositol. Uncharacterized insect transporters, predicted to be specific for glucose (based on the presence of a QLS motif) are shown in red text. Asterisks indicate identical residues in all sequences; colon indicates conservative amino acid substitution; dot marks semi-conservative amino acid substitution.

**Table S1. Summary of annotated *A. pisum* sugar porter family transporters.**

Notes: ^1^*A. pisum* sugar transporter (ApST) identification number as described in [1]; ^2^*A. pisum* genomic scaffold identification number from assembly Acyr_2.0 (NCBI accession number: ABLF00000000.2); ^3^Transmembrane (TM) helices predicted by TMHMM (available at, http://www.cbs.dtu.dk/services/TMHMM/); ^4^TIGRFAMS major facilitator superfamily (MFS) transporter, sugar porter (SP) family signature (TIGR00879) e-value scores [2]. Alternative splice forms of ApST genes are shown in light grey.

**Table S2. *de novo* identification of sugar porter (SP) family transporters [transporter classification number (T.C #) 2.A.1.1] in insect genomes**

Notes: Sugar porter (SP) family transporters (TIGRFAMS motif: TIGR00879 [2]) were identified in insect reference protein datasets (available from http://ensemblgenomes.org/) using HMMER 3.0 motif searches [3]. All identified transporters have a sequence score >237.80 (trusted cutoff).

**Table S3. Summary of annotated *A. pisum* solute:sodium symporter (SSS) family transporters.**

^1^Transmembrane (TM) helices predicted by TMHMM (available at, http://www.cbs.dtu.dk/services/TMHMM/); ^2^Pfam E-value score for sodium:solute symporter signature PF00474; ^3^ Sequence information of top BLASTP hit, aphid transporter versus

transporter classification database (TCDB). Alternative splice forms are shown in light grey.

**Table S4. Quantitative PCR primers for *Acyrthosiphon pisum* sugar transporters and housekeeping gene glyceraldehyde-3-phosphate dehydrogenase (*GAPDH*).**

Notes: Due to high sequence similarity between closely related sugar transporters (shaded light grey) it was not possible to design gene specific primers. Primers were designed to co-amplify ^1^ApST16 and ^1^ApST17 and co-ampify ^2^ApST21 and ^2^ApST28.

**Table S5. *A. pisum* sugar transporter coding sequence primers for *Saccharomyces cerevisiae* expression constructs.**

Notes: Restriction enzyme (RE) sites for forward (fwd) and reverse (rev) primers are underlined in primer sequence and Kozak translation initiation sequences [4] are shown in bold.

**References**

1. Price DRG, Tibbles K, Shigenobu S, Smertenko A, Russell CW, Douglas AE, Fitches E, Gatehouse AMR, Gatehouse JA: **Sugar transporters of the major facilitator superfamily in aphids; from gene prediction to functional characterization**. *Insect Molecular Biology* 2010, **19**:97-112.

2. Haft DH, Loftus BJ, Richardson DL, Yang F, Eisen JA, Paulsen IT, White O: **TIGRFAMs: a protein family resource for the functional identification of proteins**. *Nucleic Acids Research* 2001, **29**(1):41-43.

3. Eddy SR: **Profile hidden Markov models**. *Bioinformatics* 1998, **14**(9):755-763.

4. Kozak M: **Structural features in eukaryotic mRNAs that modulate the initiation of translation**. *Journal of Biological Chemistry* 1991, **266**(30):19867-19870.
